# Prehospital lung ultrasound for the diagnosis of cardiogenic pulmonary oedema: a pilot study

**DOI:** 10.1186/s13049-016-0288-2

**Published:** 2016-08-02

**Authors:** Christian B. Laursen, Anja Hänselmann, Stefan Posth, Søren Mikkelsen, Lars Videbæk, Henrik Berg

**Affiliations:** 1 Department of Respiratory Medicine, Odense University Hospital, Sdr. Boulevard 29, 5000 Odense, Denmark; 2 Clinical Institute, University of Southern Denmark, Odense, Denmark; 3 Department of Cardiology, Odense University Hospital, Odense, Denmark; 4 Department of Emergency Medicine, Odense University Hospital, Odense, Denmark; 5 Mobile Emergency Care Unit, Odense University Hospital, Odense, Denmark

**Keywords:** Ultrasound, Lung, Heart failure, Pulmonary oedema, Prehospital, Sensitivity and specificity

## Abstract

**Background:**

An improved prehospital diagnostic accuracy of cardiogenic pulmonary oedema could potentially improve initial treatment, triage, and outcome. A pilot study was conducted to assess the feasibility, time-use, and diagnostic accuracy of prehospital lung ultrasound (PLUS) for the diagnosis of cardiogenic pulmonary oedema.

**Methods:**

A prospective observational study was conducted in a prehospital setting. Patients were included if the physician based prehospital mobile emergency care unit was activated and one or more of the following two were present: respiratory rate >30/min., oxygen saturation <90 %. Exclusion criteria were: age <18 years, permanent mental disability or PLUS causing a delay in life-saving treatment or transportation. Following clinical assessment PLUS was performed and presence or absence of interstitial syndrome was registered. Audit by three physicians using predefined diagnostic criteria for cardiogenic pulmonary oedema was used as gold standard.

**Results:**

A total of 40 patients were included in the study. Feasibility of PLUS was 100 % and median time used was 3 min. The gold standard diagnosed 18 (45.0 %) patients with cardiogenic pulmonary oedema. The diagnostic accuracy of PLUS for the diagnosis of cardiogenic pulmonary oedema was: sensitivity 94.4 % (95 % confidence interval (CI) 72.7–99.9 %), specificity 77.3 % (95 % CI 54.6–92.2 %), positive predictive value 77.3 % (95 % CI 54.6–92.2 %), negative predictive value 94.4 % (95 % CI 72.7–99.9 %).

**Discussion:**

The sensitivity of PLUS is high, making it a potential tool for ruling-out cardiogenic pulmonary. The observed specificity was lower than what has been described in previous studies.

**Conclusions:**

Performed, as part of a physician based prehospital emergency service, PLUS seems fast and highly feasible in patients with respiratory failure. Due to its diagnostic accuracy, PLUS may have potential as a prehospital tool, especially to rule out cardiogenic pulmonary oedema.

## Background

Lung ultrasound (LUS) has over the last 20 years undergone a rapid development. From the viewpoint that the lung was an organ that ultrasound could not be used to investigate, several studies have shown that LUS can be used for a variety of the most common diagnosis seen in emergency medicine and traumatology [1, 2]. The prehospital physician faces the difficult task of differentiating between pulmonary and cardiac causes of acute respiratory failure using only history and physical examination [3]. One way to distinguish between cardiac and pulmonary causes of dyspnoea is to diagnose the presence of pulmonary oedema. Cardiogenic pulmonary oedema can with LUS be visualized as a characteristic pattern, the interstitial syndrome (IS) [1, 4, 5]. Other conditions such as non-cardiogenic pulmonary oedema, interstitial lung disease, acute respiratory distress syndrome, interstitial pneumonia can however also present itself as IS [1]. In emergency settings cardiogenic pulmonary oedema is however the most common cause of non-traumatic respiratory failure, the finding of IS has thus pragmatically been interpreted as being consistent with cardiogenic pulmonary oedema [4, 6]. This pragmatic assumption has been validated in an emergency department setting [5, 7]. Using this assumption LUS has been shown to have a high diagnostic accuracy for the diagnosis of cardiogenic pulmonary oedema which surpasses history taking, clinical examination, blood samples and chest x-ray [5]. Prehospital LUS (PLUS) to diagnose and exclude pulmonary oedema is partly described in a single case report and partly in a single prospective observational study [8, 9]. Additionally PLUS has been assessed as a possible monitoring tool for prehospital treatment of cardiogenic pulmonary oedema [10]. The results suggest that PLUS have a high diagnostic accuracy for the diagnosis of cardiac pulmonary oedema. Based on the above, some of the potential benefits of PLUS for assessing patients with acute respiratory failure are an increased prehospital diagnostic accuracy of cardiogenic pulmonary oedema, improved prehospital treatment of these patients, and earlier and more accurate notification of the receiving facility. A prospective pilot study was conducted with the primary aim to assess the feasibility of prehospital LUS in patients with signs of respiratory failure. Secondary aims were to assess time-use, and diagnostic accuracy for cardiogenic pulmonary oedema.

## Methods

### Setting and study population

The study was conducted as a prospective cross-sectional study of patients with respiratory failure in which Mobile Emergency Care Unit (MECU) in Odense, Denmark, was activated. The MECU in Odense consists of a rapid-response car operating all year round, 24/7. The car is manned with a specially trained emergency medicine technician and a physician with specialist training in anaesthesiology. Twelve physicians are employed at the MECU. The MECU operates as part of a two-tiered system, in which it supplements ambulances manned with either two emergency medicine technicians or an emergency medicine technician and a paramedic. The MECU covers an area of approximately 2500 square km and serves a population of 250,000 to 490,000 depending on time of the day. In average the MECU is dispatched to 4900 calls per year, corresponding to 13.5 calls per day. Patients with respiratory symptoms admitted to hospital by the MECU are either transported to the department of general emergency medicine or the department of cardiology, both located at Odense University Hospital, Denmark.

### Inclusion and exclusion criteria

Patients were included if they fulfilled one or more of the following two signs of respiratory failure: (1) respiratory rate >30 breaths per minute or (2) peripheral oxygen saturation without supplementary oxygen treatment <90 %. Patients were excluded if they were below 18 years of age, if the treating physician deemed that PLUS would delay life-saving treatment or transportation, or if informed consent could not be obtained due to permanent mental disability.

### Initial prehospital presumptive diagnosis and treatment

The result of the initial prehospital assessment including presumptive diagnosis and treatment initiated were prehospitally registered, encrypted and stored using an iPad. The data from the iPad were continuously transferred to a separate study database.

### Prehospital lung ultrasound examination

PLUS was performed using a SonoSite Edge (Bothell, Washington, USA) ultrasound machine, with a microconvex transducer (C11X)(8-5 mHz). As soon as the patient had been included in the project, PLUS was performed. The prehospital physician was allowed to decide whether PLUS was made on-scene or en-route to the hospital. The PLUS examination was performed using a standardized protocol. Several different LUS protocols involving a wide range of scanning zones have been described [1]. In the present study a protocol based on a mixture of a protocol described by Volpicelli et al. for use in an emergency department setting and a protocol described by Lichtenstein et al. for use in an intensive care unit setting were used [6, 11]. The transducer was placed in two scanning zones (anterior and lateral) on each side of the patient’s chest. In each scanning zone it was then noted whether three or more B-lines were present or not. Based on the PLUS findings it was then determined whether the patient was positive or negative of having interstitial syndrome. IS was defined as the presence of three or more B-lines in a longitudinal plane between two ribs in two or more of scanning fields, bilaterally. This definition was based on the international consensus definition of IS, with the modification that the total number of scanning zones were limited to two per side, rather than four [1]. It was chosen to reduce the number of scanning zones in order to decrease time used for the examination. The results of the PLUS examination, time used, and whether the findings had altered treatment or the presumptive diagnosis were prehospitally registered using the same iPad and system as described above. Time used for PLUS was defined from the physician beginning to use the ultrasound machine (incl. preparing the machine for use) to the transducer leaving the skin of the patient after completing the examination. The department receiving the patient was informed that the patient had been included in the study but the result of the PLUS examination was blinded. Prior to the initiation of the project, a short training of all the physicians working at the physician based prehospital emergency service was performed. The training consisted of a theoretical lecture (45 min) as well as practical demonstration involving hands-on training of each physician (45 min). The physicians manning the MECU were all specialists in anaesthesiology and thus had basic competencies in ultrasound for invasive procedures such as nerve blocks and vascular access. A few of the physicians had competencies in transthoracic and transeosophageal echocardiography. Only one of the physicians were experienced in the use of lung ultrasound prior to the study.

### Echocardiography

All included patients were as a part of the study referred for echocardiography performed by a cardiologist. This was done in order to systematically identify the enrolled patients’ cardiac status and to be able to identify any patients with heart failure who had not been diagnosed as IS positive.

### Blinded audit

The presence or absence of cardiogenic pulmonary oedema was established using blinded audit. Two physicians (AH (cardiologist), SP (emergency medicine physician)) independently of each other audited the patient’s hospital stay. The two auditors used the following predefined criteria to determine whether cardiac pulmonary oedema was present or absent upon patient admission to the hospital. At least two of the following three criteria had to be met: (1) clinical signs of pulmonary oedema (e.g. increased ventilation rate, hypoxemia, bilateral crackles identified by auscultation), (2) imaging study with signs of pulmonary oedema (chest X-ray, computed tomography of the chest, LUS performed in the hospital) or (3) the patient was diagnosed with a diseases which can be complicated by pulmonary oedema (systolic heart failure as part of the acute coronary syndrome, systolic heart failure, non-systolic heart failure, hypertensive crisis, arrhythmia, heart valve disease). In case of disagreement on the presence/non-presence of cardiac pulmonary oedema between the two initial auditors, an additional audit was performed by a third auditor (LV (cardiologist)) thus making the final decision. All auditors were blinded towards the results of the PLUS examination. The predefined diagnostic criteria and audit method were based on two previously conducted studies of patients with respiratory symptoms admitted to an emergency department [7, 12].

### Statistical analysis

Descriptive statistics of demographic characteristics, medical history, and initial vital signs were performed using numbers, percentages, median and interquartile-range (IQR). Descriptive statistics of the PLUS examination and its possible clinical impact were performed including site at which PLUS was performed, time used for PLUS, proportion of patients in which PLUS had altered treatment, or the prehospital presumptive diagnosis. The PLUS feasibility was calculated as the proportion of included patients in which PLUS could be performed according to the predefined ultrasound protocol. Agreement between the two initial auditors was calculated as a proportion and using the Cohen k test. The diagnostic accuracy of the initial prehospital presumptive diagnosis and PLUS for the diagnosis of cardiogenic pulmonary oedema were calculated. These calculations were expressed as sensitivity, specificity, positive predictive value (PPV), negative predictive value (NPV), positive likelihood ratio (PLR), negative likelihood ratio (NLR), and their 95 % confidence intervals (CI). Blinded audit diagnosis was used as reference test for all calculations. Data analysis was conducted using Stata Release version 11.0 (StataCorp LP).

## Results

**Fig. 1:**
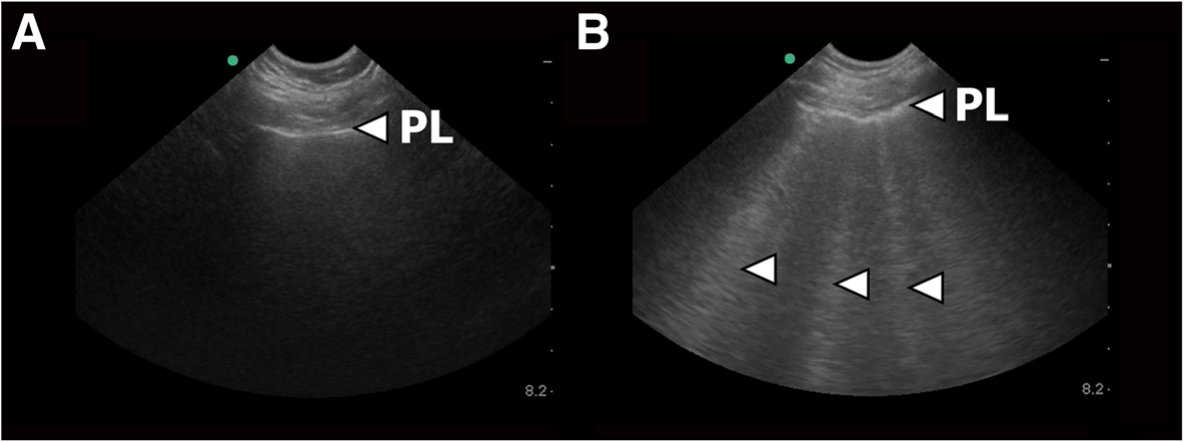
PLUS findings. **a** Image from a PLUS examination performed in a patient in which cardiogenic pulmonary oedema was absent. The pleuraline (PL) appears as a hyperechoic (*white*), horizontal line. No B-lines are present. **b** Image from a PLUS examination performed in a patient with cardiogenic pulmonary oedema. Multiple B-lines (*arrows*) are seen as hyperechoic (*white*), vertical, reverberation artefacts. The B-lines originate from the pleuraline (PL) and extends to the bottom of the field of view without fading

Between September 2013 and October 2014 a total of 45 patients were included in the study. Five patients included were excluded upon arrival at the ED were information from previous admissions revealed a diagnosis consistent with permanent mental disability. Forty patients remained for study analysis. Baseline characteristics of the included patients are provided in Table 1. Feasibility of PLUS was 100 % and median time used for the examination was 3 min (IQR 2–4 min). In all but one patient (97.5 %), the PLUS examination was performed on-scene prior to transportation of the patient [13]. The 40 PLUS examinations were performed by seven different physicians manning the MECU. In 27.5 % (95 % CI 13.0–42.0 %) of the patients PLUS findings led to a change in the prehospital presumptive diagnosis and in 22.5 % (95 % CI 9.0–36.0 %) it also led to a change in the initial prehospital treatment. Typical PLUS findings are shown in Fig. 1. The median transport time to the hospital was 10 min (IQR 5–15 min, range 4–20 min). Twenty-six (65.0 %) of the patients were admitted to the department of general emergency medicine, 13 (32.5 %) to the department of cardiology, and one (2.5 %) patient to the department of oncology. During the hospital stay, six (15.0 %) patients received non-invasive ventilation at the department of respiratory medicine and 3 (7.5 %) patients were transferred to the intensive care unit. The inhospital and 30-day mortality was 7.5 % (95 % CI 0–16.0 %) and 15 % (95 % CI 3.4–26.6 %) respectively. The most common abnormal echocardiography findings were: 14 (35 %) patients with systolic heart failure, eight (20 %) patients with mitral valve insufficiency and seven (17.5 %) patients with aortic valve stenosis. Other findings were a dilated right ventricle in five (12.5 %) patients and non-systolic heart failure in two (5 %) patients. The blinded audit diagnosed 18 (45.0 %) patients with cardiogenic pulmonary oedema. The overall agreement between the two initial auditors for the diagnosis of cardiogenic pulmonary oedema was 87.5 % (k 0.746). When stratified according to a audit diagnosis of presence or absence of cardiogenic pulmonary oedema, significantly more patients received treatment with nitroglycerin and diuretics in the cardiogenic pulmonary oedema group, whereas significantly more patients received bronchodilators and systemic steroids in the group in which cardiogenic pulmonary oedema was absent (Table 2). The contingency tables and diagnostic accuracy of the prehospital clinical assessment and PLUS for the diagnosis of cardiogenic pulmonary oedema are provided in Tables 3 and 4. No adverse events related to the PLUS examination were observed.

**Table 1:** Base-Line Characteristics of the Patients

Characteristic	(*n* = 40)
Age - years	
- Median (IQR^a^)	74 (67–81)
- Range	56–95
Sex – no. (%)	
- Male	14 (35.0)
Smoking status – no. (%)	
- Never smoked	5 (12.5)
- Current smoker	9 (23.5)
- Previous smoker	22 (55.0)
- Unknown status	4 (10.0)
Medical history – no. (%)	
- COPD	21 (52.5)
- Asthma	1 (2.5)
- Interstitial lung disease	3 (7.5)
- Coronary artery disease	13 (32.5)
- Heart failure	4 (10.0)
- Valvular heart disease	4 (10.0)
- Arterial hypertension	19 (47.5)
- Thromboembolic disease	4 (10.0)
- Diabetes mellitus	8 (20.0)
- Stroke	5 (12.5)
- Current or previous malignancy	10 (25.0)
Medication at admission – no. (%)	
- Inhaled bronchodilators	20 (50.0)
- Inhaled corticosteroids	16 (40.0)
- Oral corticosteroids	7 (17.5)
- Aspirin	11 (27.5)
- Clopidogrel	3 (7.5)
- Persantin	3 (7.5)
- Anticoagulants	10 (25.0)
- Beta-blockers	8 (20.0)
- ACE inhibitors^b^	12 (30.0)
- Calcium-channel blockers	12 (30.0)
- Diuretics	22 (55.0)
- Digoxin	5 (12.5)
- Amiodarone	2 (5.0)

^a^Interquartile range (IQR) expressed as the 25 and 75^th^

^b^ACE inhibitors denotes angiotensin-converting-enzyme inhibitor and angiotensin receptor blocker

**Table 2:** Initial prehospital vital signs and treatment stratified according to the presence or absence of cardiogenic pulmonary oedema

Initial prehospital vital signs	CPO present^a^ (*n* = 18)	CPO absent^b^ (*n* = 22)
Respiratory rate - breaths per minute		
- Median (IQR^c^)	36 (32–42)	32 (30–36)
- Range	24–50	28–42
Saturation prior to oxygen treatment - %		
- Median (IQR^c^)	80 (71–91)	85 (64–91)
- Range	54–98	50–98
Systolic blood pressure - mmHg		
- Median (IQR^c^)	161 (130–210)	155 (120–177)
- Range	84–230	80–208
Diastolic blood pressure - mmHg		
- Median (IQR^c^)	110 (80–121)	80 (66–100)
- Range	63–130	48–124
Heart rate – beats per minute		
- Median (IQR^c^)	120 (112–150)	115 (91–134)
- Range	83–180	28–143
Glasgow Coma Score		
- Median (IQR^c^)	15 (15–15)	15 (15–15)
- Range	15	13–15
Prehospital treatment administered – no. (%) (95 % CI)		
Nitroglycerin	15 (83.3 %)	3 (13.6 %)
	(64.3–100 %)	(0–29.2 %)
Diuretic	16 (88.9 %)	7 (31.8 %)
	(72.8–100)	(10.7–53.0 %)
Opioid	5 (27.8 %)	3 (13.6 %)
	(4.9–50.7 %)	(0–29.2 %)
Amiodarone	2 (11.1 %)	0 (0 %)
	(0–27.2 %)	(0–13.6 %)
Acetylsalicylic acid	4 (22.2 %)	0 (0 %)
	(0.9–43.5 %)	(0–13.6 %)
Low molecular weight heparin	1 (5.6 %)	0 (0 %)
	(0–17.3 %)	(0–13.6 %)
Bronchodilator	3 (16.7 %)	17 (77.3 %)
	(0–35.7 %)	(58.3–96.2 %)
Systemic steroid	2 (11.1 %)	14 (63.4 %)
	(0–27.2 %)	(41.8–85.5 %)
Fluid resuscitation	1 (5.6 %)	2 (9.1 %)
	(0–17.3 %)	(0–22.1 %)

^a^Result of audit was presence of cardiogenic pulmonary oedema

^b^Result of audit was absence of cardiogenic pulmonary oedema

^c^Interquartile range (IQR) expressed as the 25 and 75^th^

**Table 3:** Contingency tables

		Audit		
		CPO^a^ Present	CPO^a^ absent	Total
A				
Clinical	CPO^a^ present	14	4	18
Assessment	CPO^a^ absent	4	18	22
	Total	18	22	40
B				
PLUS	IS present	17	5	22
	IS absent	1	17	18
	Total	18	22	40

^a^ *CPO* cardiogenic pulmonary oedema

**Table 4:** Diagnostic accuracy for the diagnosis of cardiogenic pulmonary oedema

	Sensitivity (95 % CI)	Specificity (95 % CI)	PPV^a^ (95 % CI)	NPV^b^ (95 % CI)	PLR^c^ (95 % CI)	NLR^d^ (95 % CI)
Clinical assessment	77.8 % (52.4–93.6 %)	81.8 % (59.7–94.8 %)	77.8 % (52.4–93.6 %)	81.8 % (59.7–94.8 %)	4.28 (1.7–10.7)	0.272 (0.112–0.659)
PLUS	94.4 % (72.7–99.9 %)	77.3 % (54.6–92.2 %)	77.3 % (54.6–92.2 %)	94.4 % (72.7–99.9 %)	4.16 (1.91–9.05)	0.072 (0.011–0.49)

^a^ *PPV* positive predictive value

^b^ *NPV* negative predictive value

^c^ *PLR* positive likelihood ratio

^d^ *NLR* negative likelihood ratio

## Discussion

In a population of patients with respiratory failure, PLUS was fast, highly feasible, and had an acceptable diagnostic accuracy for the diagnosis of cardiogenic pulmonary oedema. Especially the sensitivity of PLUS is high, making it an excellent tool for ruling-out cardiogenic pulmonary oedema, in the case of a PLUS examination with no signs of IS. The specificity was however somewhat lower and not better than the specificity of the clinical assessment. A single prehospital study and several studies conducted in an in-hospital setting have found LUS to have both a high sensitivity and specificity for the diagnosis of cardiogenic pulmonary oedema [5, 8, 11]. One possible explanation for the lower specificity found in this study might be the relatively high proportion of patients with other conditions which may also cause IS, as reflected by the proportion of patients in which the initial chest x-ray performed in the ED was described with pulmonary fibrosis or possible interstitial lung disease (Appendix, Table 6). Another possible reason for the lower specificity might be the limited level of training in PLUS prior to the study. Studies assessing the learning curve for focused LUS have however found LUS to have a step learning curve for the diagnosis of IS with a high diagnostic accuracy despite limited training [14, 15]. A third possible explanation may be the gold standard missing patients with cardiogenic pulmonary oedema since chest x-ray often was the only imaging modality used for pulmonary assessment in the patients. Several studies have found focused LUS to have a higher diagnostic accuracy than conventional chest x-ray for the diagnosis of cardiogenic pulmonary oedema [1, 5]. Additionally, in the time gap between PLUS and initial imaging at the hospital, the patient in most cases would have received treatment and thereby reduce the severity of the cardiogenic pulmonary oedema and thus the changes which could be visualized by chest x-ray in the ED. The transportation times were however relatively short and the diagnostic criteria used for the gold standard did not solely rely on imaging findings. Based on the k value, the agreement between the two auditors was substantial according to Fleiss’ guidelines [16]. The PLUS diagnostic criteria were based on the international definition of IS, using a definition in which multiple B-lines had to be present in all four scanning zones or in both of the two anterior zones might have improved the PLUS specificity [1]. As indicated in other studies, such a definition would however also have lead to a decreased sensitivity and thereby also affect PLUS’ utility as an efficient rule-out tool [17]. The PLUS protocol assessing two zones on each hemithorax was chosen in order to reduce the time used for the ultrasound examination. The median time use of 3 min corresponds to what has been reported using a three zone approach in an intensive care setting and the eight zone approach in an ED setting [6, 11]. The two zone approach did thus not seem to reduce ultrasound examination time when compared to a three or eight zone approach. This may however be due to fact that relatively experienced or specially trained physicians performed the LUS examinations in the other studies [6, 11]. A direct comparison of the time used for the different approaches performed by the same physicians is thus needed to assess whether there is a clinical significant time difference or not. When compared to the diagnostic accuracy of the prehospital clinical assessment, the clinical impact of using PLUS as a standard diagnostic test in patients with respiratory failure would be a fast and efficient means of ruling-out cardiogenic pulmonary oedema. This would have clinical important applications regarding several aspects. The use of inhaled bronchodilators in patients with cardiogenic pulmonary oedema is associated with a worse outcome [18]. In areas with long transportation times to the nearest hospital, PLUS could potentially be used to monitor treatment response during the transportation [10]. Prehospital ultrasound of trauma patient seems to have a potential effect on prehospital triage and selection of receiving facility [19]. PLUS could possible in the same manner be used to guide whether the patient should be transferred to a cardiology department or a general emergency department. PLUS may thus potentially improve be prehospital treatment, monitoring and transportation in patients with acute respiratory failure. Depending on how the prehospital and in-hospital systems are organised, PLUS can be used to guide which patients who do not have cardiogenic pulmonary oedema and can thus safely be transported to a general emergency department and to identify patients with a high risk of cardiogenic pulmonary oedema needing initial assessment by a cardiologist.

### Limitations

Being a single-MECU study, the results cannot necessarily be applied to other MECUs or other prehospital settings. Being a pilot study with a relatively small sample size, the results has to be interpreted with caution, as also reflected by the relatively wide 95 % CI’s of the results. The study results are however still useful for generating hypotheses for future studies. The physicians performing the examinations only had limited PLUS training, this would however reflect a “real-life-setting” in which the MECU physician does not necessarily have extensive skills and experience in focused ultrasound. In some of the few published LUS learning studies, using LUS to assess whether interstitial syndrome is present or absent, a high diagnostic accuracy could be achieved even after short training as used in the present study [14, 15, 20]. Even though patients were included prospectively, the study results may have been affected by selection bias. No registration was performed of the patients not included in the study; hence no data are available for the screening process for patient participation in the study. Since patients had to be able to give informed consent in order to participate in the study, patients with very severe respiratory failure who could not give consent was not included. Seen from a study design perspective, ideally all patients with signs of respiratory failure should have been included no matter whether consent could be given or not. Such a design was however not possible in order to obtain approval from the Committee on Biomedical Research Ethics. To what extent patients were not included due to inclusion criteria not being met, due to the treating physician deemed that PLUS would delay life-saving treatment or transportation, or due to patient not being able to give informed consent is not known. Even though all the physicians received the same training prior to the study, differences in ultrasound competencies prior to the study might also have introduced selection bias if more experienced sonographers were more likely to include patients. Based on the average number of MECU activations per year and the relatively common occurrence of patients with respiratory failure one would expect that inclusion of 40 patients to the study could have been completed faster than it did. There is thus a high risk of selection bias being introduced in the patient recruitment process. To what extent this has affected study results are unknown due to the lack of available data.

## Conclusion

Performed as a part of a physician based MECU, PLUS seems fast and highly feasible in patients with respiratory failure. Due to its diagnostic accuracy, PLUS may have potential as a prehospital tool, especially to rule out cardiogenic pulmonary oedema. Due to the relatively small sample size, the results has to be interpreted with caution, the results are however still useful for generating hypotheses for future studies.

## Appendix

**Table 5:** STARD checklist for reporting of studies of diagnostic accuracy (*version January 2003*)

Section and Topic	Item #		On page #
TITLE/ABSTRACT/KEYWORDS	1	Identify the article as a study of diagnostic accuracy (recommend MeSH heading ‘sensitivity and specificity’).	p. 4
INTRODUCTION	2	State the research questions or study aims, such as estimating diagnostic accuracy or comparing accuracy between tests or across participant groups.	p. 6
METHODS			
*Participants*	3	The study population: The inclusion and exclusion criteria, setting and locations where data were collected.	p. 6–7
	4	Participant recruitment: Was recruitment based on presenting symptoms, results from previous tests, or the fact that the participants had received the index tests or the reference standard?	p. 6
	5	Participant sampling: Was the study population a consecutive series of participants defined by the selection criteria in item 3 and 4? If not, specify how participants were further selected.	p. 6–7
	6	Data collection: Was data collection planned before the index test and reference standard were performed (prospective study) or after (retrospective study)?	p. 6
*Test methods*	7	The reference standard and its rationale.	p. 9
	8	Technical specifications of material and methods involved including how and when measurements were taken, and/or cite references for index tests and reference standard.	p. 7–9
	9	Definition of and rationale for the units, cut-offs and/or categories of the results of the index tests and the reference standard.	p. 7–9
	10	The number, training and expertise of the persons executing and reading the index tests and the reference standard.	p. 7–9
	11	Whether or not the readers of the index tests and reference standard were blind (masked) to the results of the other test and describe any other clinical information available to the readers.	p. 7–9
*Statistical methods*	12	Methods for calculating or comparing measures of diagnostic accuracy, and the statistical methods used to quantify uncertainty (e.g. 95 % confidence intervals).	p. 9–10
	13	Methods for calculating test reproducibility, if done.	%
RESULTS			
*Participants*	14	When study was performed, including beginning and end dates of recruitment.	p. 10
	15	Clinical and demographic characteristics of the study population (at least information on age, gender, spectrum of presenting symptoms).	Table 1
	16	The number of participants satisfying the criteria for inclusion who did or did not undergo the index tests and/or the reference standard; describe why participants failed to undergo either test (a flow diagram is strongly recommended).	p. 10
*Test results*	17	Time-interval between the index tests and the reference standard, and any treatment administered in between.	p. 9 + Table 2
	18	Distribution of severity of disease (define criteria) in those with the target condition; other diagnoses in participants without the target condition.	Table 2
	19	A cross tabulation of the results of the index tests (including indeterminate and missing results) by the results of the reference standard; for continuous results, the distribution of the test results by the results of the reference standard.	Table 3
	20	Any adverse events from performing the index tests or the reference standard.	p. 11
*Estimates*	21	Estimates of diagnostic accuracy and measures of statistical uncertainty (e.g. 95 % confidence intervals).	p. 10–11 Tables 2 + 4,
	22	How indeterminate results, missing data and outliers of the index tests were handled.	No missing data
	23	Estimates of variability of diagnostic accuracy between subgroups of participants, readers or centers, if done.	Not done
	24	Estimates of test reproducibility, if done.	Not done
DISCUSSION	25	Discuss the clinical applicability of the study findings.	p. 12–14

**Table 6:** Initial chest x-ray findings

Chest X-ray finding – no. (%) (95 % CI)	(*n* = 34)
Interstitial fibrosis	16 (47.1) (29.4–64.7)
Pulmonary oedema	15 (44.1) (26.5–61.7)
Enlarged heart	13 (38.2) (21.0–55.4)
Emphysema	8 (23.5) (8.5–38.6)
Unilateral pulmonary opacity	7 (20.6) (6.3–34.9)
Bilateral pulmonary opacities	5 (14.7) (2.2–27.2)
Unilateral pleural effusion	4 (11.8) (0.4–23.1)
Bilateral pleural effusion	3 (8.8) (0–18.9)
Widened mediastinum	3 (8.8) (0–18.9)
